# Inflammatory Mediators of Hepatic Steatosis

**DOI:** 10.1155/2010/837419

**Published:** 2010-03-16

**Authors:** Elizabeth Hijona, Lander Hijona, Juan I. Arenas, Luis Bujanda

**Affiliations:** ^1^Gastroenterology Department, Donostia Hospital, CIBERehd, University of the Basque Country, 20010 San Sebastián, Spain; ^2^Gastroenterology Department, Basurto Hospital, 48013 Bilbao, Spain

## Abstract

Nonalcoholic fatty liver disease (NAFLD) is rapidly becoming a world-wide public health problem. NAFLD represents a spectrum of disease ranging from “simple steatosis”, which is considered relatively benign, to nonalcoholic steatohepatitis and to NAFLD-associated cirrhosis and end-stage liver disease. The etiology of NAFLD and its progression is complex and remains incompletely understood. The progression of the disease involves many factors. Apart from the two hits, the accumulation of TG and the development of fibrosis and necroinflammatory processes, exit numerous molecules associated with these two hits. Among them we can highlight the pro-inflammatory molecules and adiponectins. This review focuses on the growing evidence from both experimental and human studies suggesting a central role of cytokines in the pathogenesis of NAFLD. We review the role of cytokines as key regulators of insulin sensitivity and hepatic lipid overloading, liver injury and inflammation, and fibrosis with an emphasis on potential therapeutic implications.

## 1. Introduction

Nonalcoholic fatty liver disease (NAFLD) is a chronic inflammatory disease involving a wide range of disorders: from simple steatosis; through steatohepatitis, fibrosis and cirrhosis; to hepatocarcinoma. Historically, steatosis has been considered a benign disease. However, it is a necessary requirement for the development of NAFLD, characterized by the accumulation of lipids in the cytoplasm of hepatocytes. Its prevalence is not well known, but it is estimated to be higher than 3%. It is a complex disease in which many factors play a role including obesity, insulin resistance, and oxidative stress, among others.

Nonalcoholic fatty liver disease (NAFLD) is characterized by liver damage similar to that caused by alcohol, but which occurs in individuals that do not consume toxic quantities of alcohol [1–10]. The prevalence of NAFLD is not well known, but according to various studies, ranges between 3% and 24% [11–17]. It is likely that its prevalence will increase in the future, due to an increase in the prevalence of being overweight and obesity [18–21].

## 2. Etiology

NAFLD has been associated with many etiological factors [1, 2, 22], the most common ones being obesity, type 2 diabetes mellitus, and dyslipidemia. In most series, one, two, or three factors occur in 80% and 95% of individuals. The association between diabetes or intolerance to glucose and two or more of the following clinical signs: obesity, hyperlipidemia, high blood pressure, and hypoalbuminemia is known as metabolic syndrome X [18, 21, 23]. This syndrome is also associated with NAFLD.

Obesity (>10% of normal weight or Body Mass Index >30) (BMI) is the most common cause of NAFLD: approximately 80% of patients are obese, and the opposite is also true, 80% obese people suffer from NAFLD [24–27]. 

Obesity and being overweight are problems of growing importance in developed societies due to their prevalence, not only in adults but also in children [28, 29]. They cause several metabolic and associated disorders, such as resistance to insulin, diabetes, and dyslipidemia [30]. Obesity is also known to be a risk factor for chronic diseases such as: diabetes, high blood pressure, heart and cerebrovascular diseases, and some types of cancer [31–33].

## 3. Pathogenesis

The exact pathogenic mechanisms involved are still not well known. However, available evidence has enabled a tenable theory of pathogenesis to be proposed involving two stages, known as “two-hit theory” [4, 34–37]. 

### 3.1. Two Hits Theory

The first hit of steatosis, giving rise to the first lesions is caused by excess free fatty acids (FFA) in the liver, which are sterified to triglycerides (TG) [38, 39]. These initial lesions make the liver vulnerable to aggressive factors of the second hit, which is caused by the oxidative stress and proinflammatory cytokines (TNF-*α*, TGF-beta, IL-6, IL-8). This leads to the occurrence of lesions in the hepatocytes, inflammation and fibrosis, and consequently the evolution of hepatic steatosis to steatohepatitits. Some poorly understood genetic factors may explain whether steatosis evolves to steatohepatitis or not [16, 34, 36, 37, 39–41].

#### 3.1.1. First Hit

A feature of obesity, type 2 diabetes, hyperlipidemia and metabolic syndrome X is resistance to insulin [36, 42–44]. Adipocyte insensitivity to insulin inhibits the regulation of the lipase in the adipose tissue, and large quantities of free fatty acids (FFAs) are released [41, 45–48]. Oversupply of FFAs to the liver is the main mechanism that leads to steatosis in these patients. However there are also other mechanisms. One of these is an increase of insulinemia, whether or not caused by insulin resistance, which inhibits the carnitine palmitoyltransferase enzyme, and this reduces the mytochondria beta-oxidation of FFAs [5, 49–51]. Hyperinsulinism also decreases the synthesis of apolipoprotein B-100 in the liver, which causes a decrease in the secretion of very low density lipoproteins (VLDL) [52, 53]. Finally, excess production of exogenous and endogenous glucose (in obesity and diabetes respectively), together with hyperinsulinemia, increase the synthesis of FFAs in the liver. The final result is a positive FFA balance, from oversupply and/or failure in lipid, leading to accumulation in the liver. It is not well understood why not all patients with risk factors (obesity, type 2 diabetes mellitus, hyperlipidemia) develop NAFLD. Recently, it has been demonstrated that patients with NAFLD have an increased prevalence of polymorphism in TNF-*α* 238 (TNFA allele), inducing an overexpression of TNF-*α* in adipose tissue, and this, in turn, disrupts insulin receptors, causing resistance to insulin [53–55]. The transcription factor HNF3*α* (hepatocyte nuclear factor) is an important target for research. Its presence is related to the inhibition of the accumulation of fatty acids. If overexpressed, this transcription factor triggers a reduction in the synthesis of fatty acids; that is, it has an opposite effect to steatosis [56–58].

#### 3.1.2. Second Hit

FFAs cause an increase in the expression of cytochrome P-450 2E1 (CYP 2E1). This is a microsomal enzyme that takes part in the beta-oxidation of long and very long chain FFAs, causing the production of reactive oxygen metabolites [59]. On the other hand, some long-chain FFAs are metabolized by peroxisomal beta-oxidation. This oxidation generates hydrogen peroxide, that produces hydroxyl radicals in the presence of iron, both being also reactive oxygen metabolites [60]. The excess of reactive oxygen metabolites depletes natural antioxidants such as glutathione and vitamin E in the liver, causing oxidative stress resulting in lipid peroxidation [26, 35, 39]. In turn, this causes damage in the hepatocyte organelles and membranes, leading to degeneration and hepatocellular necrosis [26]. The damage caused by lipid peroxidation in mitochondria, apart from changing their morphology (megamitochondria), distorts the transfer of electrons in the respiratory chain and this results in more production of reactive oxygen metabolites, closing the cycle by causing more oxidative stress [7, 16, 61–63]. 

Oxidative stress activates the Fas ligand and nuclear factor kappa *β* (NF-kappa *β*). The former causes degeneration and hepatocyte death, and the latter stimulates the synthesis of proinflammatory cytokines (TNF-*α*, TGF-*β*, IL-8) [22]. In addition, the final products of lipid peroxidation, malonaldehyde and 4-hydroxynonenal have chemotactic properties, activating proinflammatory cytokines (TNF-*α*, TGF-*β*, IL-6, IL-8) and stimulating hepatic collagen-producing stellate cells. The end result is a mixed lesion, known as steatohepatitis, characterized by degeneration and hepatocyte necrosis, inflammatory infiltrate and fibrosis, as well as steatosis [1, 22]. Malonaldehyde and 4-hydroxynonenal are also covalently bound to proteins and produce protein clusters with antigenic properties. Secondarily, antibodies may appear, which are able to cause immune-mediated hepatocellular injury (autoimmune hepatitis) [64]. One of these protein inclusions corresponds to Mallory's hyaline [64]. Ongoing oxidative stress and lipid peroxidation results in the continued production of collagen leading to fibrosis reaching the stage of hepatic cirrhosis [1, 36]. 

The passage of endotoxins from the intestine to the splenic circulation causes portal endotoxemia. Endotoxins stimulate the synthesis of proinflammatory cytokines in the liver [36]. This mechanism is essential in the development of steatohepatitis associated with intestinal bypass, since this type of surgery encourages bacterial overgrowth and endotoxemia from the dysfunctionalised loop [36]. Obese patients have intense intrahepatic expression of the enzyme nitric oxide synthase [26], which is induced by endotoxins and TNF-*α*; furthermore, obese mice have been found to suffer from hepatic hypersensitivity which causes them to develop more severe degrees of steatohepatitis [4]. 

In this complex context, the role of several molecules are involved.

**Leptin**

Leptin is an adipocyte-secreted, negative feedback hormone that acts on the hypothalamus to regulate both food intake and energy expenditure [65–67]. Leptin levels are elevated in obesity [68]. It is believed that leptin has a lipostatic function: when the quantity of fat stored in the adipocytes increases, leptin is released into the bloodstream. This constitutes a negative feedback signal to the hypothalamus, informing the hippocampus that the body has enough food and the appetite should be reduced. When the adipose tissue mass increases a certain level beyond equilibrium, there is an increase of the synthesis and secretion of leptin, triggering several compensating effects in the hypothalamus: a decrease in appetite by the production of anorexigenic peptides (inducing loss of appetite) and the suppression of orexigenic peptides; an increase in energy expenditure by increasing of the basal metabolism and body temperature; and also a change in the equilibrium levels of hormones to reduce lipogenesis (production of fats) and to increase lipolisis (use of the body fat stored to produce energy) in the adipose tissue. The regulation of the secretion of leptin takes place on long timescales, mainly due to variations in body mass and stimulating effects of insulin. Note however that many obese people have high concentrations of leptin in serum, or resistance to leptin, suggesting that other molecules such as ghrelin, serotonin, cholecystokinin, and the neuropeptide Y also have an effect in the sensation of satiety and contribute to body mass regulation. Specifically, the secretion of leptin is correlated to body mass. It prevents the occurrence of NAFLD, indirectly though the central neural pathway, and directly through the activation of AMPK (AMP-activated protein kinase) [69–72]. Indeed, in patients with NAFLD it has been observed that leptin levels are directly correlated to the severity of the disease.

Leptin deficient ob/ob mice show markedly reduced levels of energy expedenditure and become obese even when pair fed compared with littermate controls. The marked steatosis observed in this group indicates that leptin prevents fatty liver, both indirectly, through central neural pathways, and directly via hepatic activation of adenosine monophosphate-activated protein kinase (AMPK) [69–71].

In patients with NAFLD, the analysis of circulating levels of leptin, has provided results more conflicting. Leptin levels found to be increased in nonalcoholic steatohepatitis, independently of BMI, with higher in patients with advanced disease [73]. In an other study, leptin levels directly correlated with the severity of steatosis but not with inflammation or fibrosis [74]. The strong evidence for leptin as a fibrogenic agent in animal models is not clearly paralleled by evidence on circulating levels in patients [71].

**Adiponectin**

(Also known as Acrp30, AdipoQ, apM1 o GBP28), a hormone synthesized exclusively by the adipose tissue takes part in the metabolism of glucose and fatty acids. It is secreted by adipocytes and is considered as an anti-inflammatory adipokine [68]. In general, adiponectin reduces inflammation, stimulating secretion of anti-inflammatory cytokines (e.g., IL-10), blocking NF-KB activation, and inhibiting realease of TNF-*α*, IL-6 and chemokines [75]. Adiponectin concentrations inversely correlated with fat mass and are down-regulated in obesity and type 2 diabetes. Adiponectin exerts insulin-sensitizing effects in the liver, skeletal muscle, and adipose tissue. Like leptin, adiponectin regulates whole-body lipid partitioning and has hepatoprotective and antifibrogenic effect in condition of liver injury [68].

In experimental alcoholic and nonalcoholic models, the administration of adiponectin, ameliorated necroinflammation, and steatosis, partly via inhibition of TNF-*α* [76]. In obese mice, the administration of adiponectin improved liver injury, increasing PPAR- *α* and reducing TNF-*α* [77].

In patients with nonalcoholic steatohepatitis, adiponectin levels were reduced in comparison with control and simple steatosis patients [78]. Bugianesi et al. [79] found that adiponectin levels correlate with suppression of endogenous glucose production and predict the presence of the metabolic syndrome. However, adiponectin levels was inversely associated only with intrahepatic fat but not with inflammation and fibrosis. In patients with diabetes, levels of adiponectin are inversely correlated to hepatic fat content and to endogenous glucose production. This hit suggests, that adiponectin may represent a link between hepatic fat and insulin resistance (IR) [80]. Also, gentic factors produce alterations in the adiponectin levels. Polymorphisms of the adiponectin gene have been associated with higher risk of type 2 diabetes and cardiovascular disease [81].

**Interleukin-6 (IL-6)**

It has a pleiotropic action, and in animals models it has been associated with protection of steatosis [71]. It is associated with hyperinsulinaemia and IR [82].

IL-6 is overexpressed in the adipose tissue of obese patiens [71]. Increased hepatic IL-6 production may play an important role in NASH development, as well as in systemic insulin resistance and diabetes [83]. Chronically elevated IL-6 levels lead to inappropriate hyperinsulinaemia, reduced body weight, impaired insulin-stimulated glucose uptake by the skeletal muscles and marked inflammation in the liver. Thus, the pleiotrophic effects of chronically elevated IL-6 levels preclude any obvious usefulness in treating obesity or its associated metabolic complications in man, despite the fact that weight reduction may be expected [82].

**Resistin**

Resistin is a recently discovered adipokine, secreted by adipose tissue and macrophages [68].

Several rodent models shown that resistin may be a link between IR and obesity [70]. In resistin-deficient mice placed on high-fat diet, fatty infiltration and secretion of low density lipoprotein are decreased. This suggests, a role of resistin in the induction of hepatic steatosis [84].

In humans, the biology of resistin is not clearly defined. Most studies demonstrates that resistin is expressed by bone marrow [72].

## 4. Genetic Factors

Not all patients with similar risk factors (obesity, diabetes, hyperlipidemia, etc.) develop steatosis. Moreover, as with alcoholic steatosis, not all the patients with simple steatosis go on to develop the lesions characteristic of steatohepatitis nor do all patients with steatohepatitis reach the stage of cirrhosis, and those that do take widely varying times to develop the condition. It has been suggested that this range of outcomes may depend on some genetically determined factors, such as genetic polymorphism in the CYP2E1, TNF-*α*, or IL-10 promoter regions [1]. In NAFLD, these genetic factors are unknown but it has been suggested that the development of steatosis may depend on the occurrence of alterations in the genes involved in insulin resistance, or in those codifying proteins that are involved in the hepatic metabolism of lipids [85].

## 5. Future Therapeutic Targets

The basic pillars to the treatment of NAFLD include weight loss and change in lifestyle. Currently, are using different types of drugs for the treatment of non-alcoholic steatosis.Among them we highlight the antidiabetic agents such as pioglitazone, rosiglitazone, troglitazone, and metformin. Rosiglitazone and troglitazone have been retired from the market because it produced severe liver damage [86]. Metformin is the molecule being tested today, as it can reduce the steatosis [87]. On the other hand, they have used drugs with antioxidant powers as betaine, N-acetylcysteine, Vitamin E, and other drugs, that apart from its antioxidant properties, have cytoprotective properties, as ursodeoxycholic acid. Finally, they have also been used as anticitoquines drugs anti-TNF/Infliximab [88].

All these studies are very preliminary. It is necessary to test new drugs that have been able to stop steatosis. Among the new drugs being tested, we emphasize the Allopurinol [89], omega-3 fatty acids [90], bezafibrate [91], the combination of ezetimibe/simvastatin [92], SRT1720 (SIRT1 activator) [93], Viusid (nutritional supplement) [94], Pan-caspase [95], bicyclol [96] and Losartan [97] among others. It has been observed that these drugs may be promising, as they decrease injuries NAFLD. But remember that these studies have been realized in animals and are necessary human trials.

The above-mentioned molecules, represent a convincing target for the development of novel therapies in liver diseases, and adiponectins appear as the forerunner candidate.

Adiponectin concentrations are reduced in people suffering from obesity, type 2 diabetes mellitus and coronary arterial disease [71, 76, 98]. The proinflammatory cytokines TNF-*α* and IL-6 play important roles in obesity and the evolution of the disease [99–102]. Another target of interest is the constitutive androstane receptor (CAR). It improves the sensitivity to glucose and steatosis by inhibiting hepatic lipogenesis and inducin *β*-oxidation [103]. Also, the peroxisome proliferator-activated receptor (PPAR*α*) is responsible for the increase in the oxidation of fatty acids and decreases blood levels of triglycerides. In patients with steatosis, it has been observed that its levels decrease considerably [104], while those of the prolipogenic transcription factor Sterol Regulatory Element Binding Proteins (SERBP-1c) significantly increase [105]. For these reasons, PPAR*α* is also considered to be an interesting target to study in relation to lipid metabolism and obesity [104–106].

In the not too distant future, we must be able to diagnose steatosis, without using invasive methods. Today, for the diagnosis, liver biopsy is used, but it would be very interesting to be able to perform diagnosis using noninvasive techniques. For example, determining the amount of liver fat by magnetic resonance techniques. It would be very useful to perform a predictive test, but for this, we need much more research about the interaction of different factors, molecules, and genes [107]. The identification of the molecular mechanism leading to fat accumulation and oxidative imbalance in steatotic livers, as well as genome and proteome studies from patients to various stages of the disease, is expected to improve the diagnostic and therapeutic approaches. In this way, attractive pharmacological designs include new molecules which can be able to decrease lipid from livers and to improve insulin sensitivity.

The apparence of novel approaches is eagerly awaited.
